# An Updated Review on the Modulation of Carbon Partitioning and Allocation in Arbuscular Mycorrhizal Plants

**DOI:** 10.3390/microorganisms10010075

**Published:** 2021-12-30

**Authors:** Isaac A. Salmeron-Santiago, Miguel Martínez-Trujillo, Juan J. Valdez-Alarcón, Martha E. Pedraza-Santos, Gustavo Santoyo, María J. Pozo, Ana T. Chávez-Bárcenas

**Affiliations:** 1 Facultad de Biología, Universidad Michoacana de San Nicolás de Hidalgo, Morelia 58030, Mexico; isaac.salmeron@umich.mx; 2 Centro Multidisciplinario de Estudios en Biotecnología, Universidad Michoacana de San Nicolás de Hidalgo, Morelia 58880, Mexico; jose.alarcon@umich.mx; 3 Facultad de Agrobiología “Presidente Juárez”, Universidad Michoacana de San Nicolás de Hidalgo, Uruapan 60170, Mexico; martha.elena.pedraza@umich.mx; 4 Instituto de Investigaciones Químico Biológicas, Universidad Michoacana de San Nicolás de Hidalgo, Morelia 58030, Mexico; gustavo.santoyo@umich.mx; 5 Departamento de Microbiología del Suelo y Sistemas Simbióticos, Estación Experimental del Zaidín, CSIC, 18008 Granada, Spain

**Keywords:** arbuscular mycorrhiza, sucrose metabolism and translocation, lipid metabolism, translocation

## Abstract

Arbuscular mycorrhizal fungi (AMF) are obligate biotrophs that supply mineral nutrients to the host plant in exchange for carbon derived from photosynthesis. Sucrose is the end-product of photosynthesis and the main compound used by plants to translocate photosynthates to non-photosynthetic tissues. AMF alter carbon distribution in plants by modifying the expression and activity of key enzymes of sucrose biosynthesis, transport, and/or catabolism. Since sucrose is essential for the maintenance of all metabolic and physiological processes, the modifications addressed by AMF can significantly affect plant development and stress responses. AMF also modulate plant lipid biosynthesis to acquire storage reserves, generate biomass, and fulfill its life cycle. In this review we address the most relevant aspects of the influence of AMF on sucrose and lipid metabolism in plants, including its effects on sucrose biosynthesis both in photosynthetic and heterotrophic tissues, and the influence of sucrose on lipid biosynthesis in the context of the symbiosis. We present a hypothetical model of carbon partitioning between plants and AMF in which the coordinated action of sucrose biosynthesis, transport, and catabolism plays a role in the generation of hexose gradients to supply carbon to AMF, and to control the amount of carbon assigned to the fungus.

## 1. Introduction

Arbuscular mycorrhiza (AM) is a mutualistic association between fungi from the Glomeromycotina group and plants from most phylogenetic clades [1,2]. The mutualistic nature of arbuscular mycorrhizae implies a bidirectional flow of nutrients between plant roots and arbuscular mycorrhizal fungi (AMF). The plant gives up part of its photoassimilates to the fungus, an obligate symbiont with a heterotrophic metabolism that grows and develops in the internal root tissues [3]. One key benefit for the host plants from this biological interaction is the improved acquisition of water and mineral nutrients, in particular phosphorus (P) [4,5,6]. The regulation of this resource exchange between the plant and fungal partners is key for the functioning of symbiosis, as it determines the net outcome of the interaction. Major research efforts have been devoted in the last few decades to an understanding of the trading of resources between the plant and fungal symbiont [7,8].

During mycorrhizal colonization, AMF form intraradical tree-like branched hyphae structures called arbuscules, which are the main nutrient and water exchange sites between symbionts. Arbuscules develop within the cortical cells of the roots, but they do not compromise the integrity of the host cell; instead, arbuscules are surrounded by the cortical cell’s plasma membrane, forming the so-called periarbuscular membrane [9,10]. The fungus takes from the soil minerals such as P and nitrogen and releases them in the periarbuscular space, which is the interface between the periarbuscular membrane and the arbuscule. These nutrients are then imported into the plant cell by specific nutrient transporters, assisted by H+ -ATPases located in the periarbuscular membrane [11,12,13].

The presence of the fungal symbiont implies an extra demand of energy from the plant and, therefore, alters carbon distribution. It has been estimated that 4 to 23% of the total photoassimilated carbon is destined for the maintenance of symbiosis [14,15]. Indeed, significant increases in photosynthesis have been reported in mycorrhizal plants, and this increase has been associated with the fungal symbiont acting as an additional carbon sink in the root system [16]. In fact, in mycorrhized cucumber (*Cucumis sativus*), a 10 to 40% reduction in the photosynthetic capacity occurs after the removal of extraradical mycelia, denoting the sink stimulation promoted by the AMF colonization [17]. It has also been suggested that the increase in the photosynthetic rate occurs as a consequence of the improvement in nutrient acquisition, particularly phosphorus, promoted by AMF [18]. However, a meta-analysis of legumes proposed that the increased nutrient acquisition in mycorrhizal plants does not fully explain their improvement in photosynthesis; however, other plant traits promoted by the symbiosis, such as the increased harvest index (the ratio of seed production to total shoot dry matter), may increase the sink strength and act as additional stimulators of photosynthesis [19]. Moreover, the dynamics of carbon distribution in mycorrhizal plants are also determined by the genetic background of both symbionts and their developmental stage, together with the biotic and abiotic environmental factors that influence the symbiosis [12].

During photosynthesis, CO_2_ is assimilated into carbohydrates. Sucrose (Suc) is the main carbohydrate used by most plants to transport carbon from the photosynthetic (source) to the heterotrophic (sink) tissues. Suc is biosynthesized in the cytoplasm of mesophyll cells by the coupled action of Suc-P synthase (SPS; EC 2.4.1.14) and Suc-P phosphatase (SPP; EC 3.1.3.24). It has been suggested that SPS is a key enzyme and one of the rate-limiting steps controlling the carbon flux to Suc biosynthesis [20,21].

The Suc synthesized in mesophyll cells is transported to the phloem by symplastic and apoplastic pathways mediated by Suc Transporters (SUT) and Sugar Will Eventually be Exported Transporters (SWEET) [22,23]. In the phloem, Suc diffuses through mass flow until it reaches the sink tissues, where it may be the substrate to different catabolic pathways to obtain energy or be a precursor for structural or storage molecules [24]. Depending on the pathway, Suc is cleaved by Suc synthase (SuSy; EC 2.4.1.13) to produce UDP-Glucose (UDP-Glc) and Fructose (Fru), or it is hydrolyzed by invertases (EC 3.2.1.26) to yield Glucose (Glc) and Fru [25]. In mycorrhizal roots, these hexoses are transported across the periarbuscular and fungal plasma membranes in arbusculated cells to reach the fungal partner [26].

In addition to the carbon in the form of hexoses, the host plants also contribute lipids to AMF [27,28]. Lipid auxotrophy, as a metabolic property of AMF, explains their obligate biotrophy. This auxotrophy is caused by the absence of the fatty acid synthase type I (type I FAS) molecular complex that controls de novo lipid biosynthesis in fungi and other eukaryotes [29,30]. The recent characterization of the lipid acquisition by AMF in the mycorrhizae, established with bryophytes and vascular plants, revealed that the mechanisms of lipid biosynthesis and transport are conserved among land plants. This supports the hypothesis that land colonization by plants during the Ordovician was favored by mycorrhizal interactions reminiscent of the arbuscular type and primitive plants [31,32,33].

While the molecular mechanisms involved in the carbohydrate and lipid translocation from plant to fungi in mycorrhizae have not been fully unraveled, there is significant evidence related to the role of particular enzymes or transporters [34]. For example, transcripts encoding hexose transporters from a superfamily of Glc and Fru facilitators [35] and apoplastic invertases involved in Suc hydrolyzation to hexoses [36,37] have been detected in arbusculated cells [8]. Transcriptional fusions of SWEET promoters to reporter genes have also located the SWEET promoter activity in arbuscule-containing cells, and SUT proteins have been specifically located in the periarbuscular membrane by immunolocalization [8,38,39]. The WRINKLED transcription factors, the main controllers of lipid biosynthesis in plants [40] are also involved in the regulation of key genes involved in lipid transfer to the AMF [33,41,42], such as Required for Arbuscular Mycorrhization 2 (*RAM2*) encoding a glycerol-3-phosphate acyl transferase (EC: 2.3.1.15) and Stunted Arbuscule 1 and 2 (*STR1* and *STR2*), which belong to the heterodimeric Adenosine Triphosphate (ATP)-Binding Cassette (ABC) transporter family [27,43,44,45].

The goal of this review is to analyze the current state of the art on the role of carbon metabolism in mycorrhizae interactions, and to propose an integrated model of carbon partitioning during this symbiotic association.

## 2. Sucrose Metabolism in Plants

**Figure 1:**
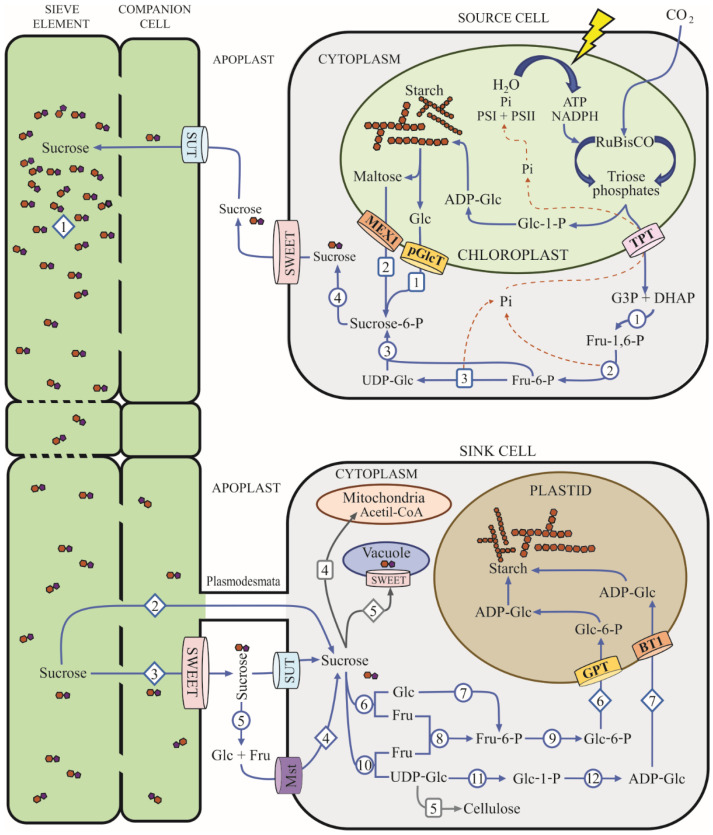
Common plant pathways of carbon assimilation and partitioning from source to sink tissues. This diagram shows a current model for organic carbon biosynthesis and translocation from photosynthetic to heterotrophic cells (based on previous models by MacNeill et al., 2017 and López-González et al., 2019 [51,52]). Blue arrows trace the current carbon flow routes, the discontinued orange arrows show Pi flux, and the colored barrels designate carbohydrate transporters. Enzymes are indicated in numbered circles as: (1) Fructose 1,6-bisphosphate aldolase (EC 4.1.2.13); (2) FBPase (EC 3.1.3.11); (3) SPS (EC 2.4.1.14); (4) SPP (EC 3.1.3.24); (5) Apoplastic invertase (EC 3.2.1.26); (6) Neutral (cytoplasmic) invertase; (7) Hexokinase (EC 2.7.1.1); (8) Fructokinase (EC 2.7.1.4); (9) Phosphoglucoisomerase (EC 5.3.1.9); (10) SuSy (EC 2.4.1.13); (11) UDPase (EC 2.7.7.9); (12) ADP-glucose pyrophosphorylase (EC 2.7.7.27) [52]. The numbers in rounded rectangles denote specific metabolic pathways: (1) and (2) starch-derived carbohydrates translocated to cytoplasm by Maltose Excess Protein (MEX1; ([52,53])) or Plastidic Glucose Translocator (pGlcT; [52,54]), as possible precursors for Suc biosynthesis; (3) synthesis of UDP-Glucose from Fructose-6-P; (4) Suc catabolism for energy metabolism; (5) Cellulose biosynthesis. Diamonds indicate potential carbon fluxes as: (1) Suc mass flow from source to sink tissues through the phloem; (2) symplastic transport of Suc from phloem to the sink cells; (3) apoplastic transport of Suc from the phloem to sink cells; (4) Monosaccharides transported from the apoplast to sink cells by Monosaccharide transporters (Mst), as potential substrates for Suc biosynthesis; (5) Vacuole import of Suc for transient storage; 6 and (7) Glc-6-P and ADP-Glc imported into plastids by specific transporters (GPT; Glc-6-P/Pi translocator and BT1; Adenine nucleotide transporter Brittle1, respectively, (see [51,52,54] for review) and directed to starch biosynthesis.

**Sucrose** is a non-reducing disaccharide synthesized in source tissues either from chloroplast carbon assimilation products in leaves, mainly triose phosphates (triose-Ps) or starch (Figure 1), or from starch accumulated in storage organs such as tubers or seeds. Triose-Ps are the first stable products of photosynthetic carbon fixation. They are formed in the chloroplasts of mesophyll cells and moved to the cytoplasm through the Triose Phosphate Translocator (TPT) located at the inner membrane of chloroplasts. The TPT is an antiporter that exports triose-Ps in exchange for inorganic phosphate (Pi) from the cytoplasm [46]. The imported Pi is later used to regenerate ATP by the light reactions of photosynthesis, while the triose-Ps are condensed to fructose-1,6-bisphosphate (Fru-1,6-BP) by fructose 1,6-bisphosphate aldolase (FBP; EC 4.1.2.13) [47]. Then, Fru-1,6-BP is dephosphorylated by fructose bisphosphatase (FBPase; EC 3.1.3.11) to render fructose-6-phosphate (Fru-6-P). Subsequently, Fru-6-P is isomerized to Glucose-6-phosphate (Glc-6-P) by phosphoglucoisomerase (PGI; EC 5.3.1.9); then Glc-6-P is isomerized by phosphoglucomutase (PGM; EC 5.4.2.2) to Glucose-1-phosphate (Glc-1-P), which is then used as a substrate of UDP-glucose pyrophosphorylase (UDPase; EC 2.7.7.9) to produce UDP-Glucose (UDP-Glc). The transfer of a glucosyl moiety from UDP-Glc to Fru-6-P is catalyzed by SPS, and the Sucrose-6-P (Suc-6-P) obtained is dephosphorylated by SPP to yield Suc as the final product [48,49,50] (Figure 1).

The metabolic pathway of Suc biosynthesis is modulated by the enzymatic activities of FBPase and SPS as the major rate-limiting steps of the pathway and photosynthate partitioning in leaves [55]. FBPase catalyzes the first irreversible reaction bearing the intermediate metabolite in Suc biosynthesis, and its enzymatic activity is also regulated by both fine and coarse control mechanisms; the rise of photosynthesis rates increases the accumulation of triose-Ps and their later translocation to the cytosol causes the increment of Fru-1,6-P, activating FBPase [56]. The accumulation of hexose phosphates in cytosol leads to the down-regulation of FBPase by the synthesis of its allosteric inhibitor fructose-2,6-bisphosphate (Fru-2,6-BP), which blocks the pathway towards Suc production [57]. Under these conditions, the concentration of Pi lowers in the cytosol; thus, triose-Ps are no longer translocated from the chloroplast [48,57].

Despite the importance of FBPase in carbon partitioning to Suc biosynthesis, the knockout of the cytosolic FBPase gene in *Arabidopsis* does not lead to strong phenotypic modifications, nor does it compromise the plant viability regardless of the carbon imbalance, which was observed as an increase in the starch content of the leaves without modification of the Suc content [58]. In cytosolic FBPase antisense potato (*Solanum tuberosum*) lines, the accumulation of starch in leaves increased compared to the wild-type, and this was more pronounced when the transgenic plants were exposed to light; however, the development of organs such as tubers and the levels of soluble sugars, including Suc, where mostly unaltered [59]. Thus, a compensatory mechanism for the carbon imbalance generated by the cytosolic FBPase mutation involves the mobilization of the starch from chloroplasts to provide hexoses as the carbon source exported to the cytosol to drive Suc biosynthesis [59,60]. This suggests that Suc biosynthesis can avoid the triose-Ps translocation and the further reaction catalyzed by FBPase (Figure 1).

The reaction catalyzed by SPS is freely reversible in vitro. However, the values of standard free energy calculated for in vivo conditions are lower than those in vitro, displacing the reversible SPS reaction from equilibrium to sucroneogenesis; this is thought to be due to the rapid removal of Pi from Suc-6-P by SPP under in vivo conditions, which suggests a close interaction between both the SPS and SPP enzymes [20,21,61]. The contribution of SPS in the control of C partitioning to Suc has been demonstrated by gene overexpression and antisense repression approaches, which resulted in alterations in Suc synthesis and allocation, and the photosynthetic capacity of the transgenic plants [57]. The pattern of expression of SPS genes is highly regulated at the tissue and developmental levels, and its enzymatic activity also responds to positive (Glc-6P) and negative (Pi) allosteric effectors, as well as to covalent modification by phosphorylation [62,63].

Once synthesized in photosynthetic tissues, Suc is transported to the phloem parenchyma cells where SWEET catalyzes the efflux of Suc into the phloem apoplast to achieve the phloem loading [23]. Then SUT uptakes Suc to the phloem, which is translocated to heterotrophic tissues by mass flow driven by the concentration gradient. Then it enters the sink tissues by symplastic routes through the plasmodesmata or by apoplastic pathways involving the participation of SWEET and SUT [22,24]. There, Suc is released into the apoplastic space, and it can be actively transported to the sink cells by plasmodesmata, or it may be previously hydrolyzed by apoplastic invertases to produce Glc and Fru that are later incorporated in the sink cells by hexose transporters [24,64] (Figure 1).

Sucrose downloaded into the sink tissues can be the substrate for different metabolic pathways to generate energy or it can act as a C-skeleton to support the biosynthesis of structural molecules, reserve polymers, or specific metabolites in response to environmental conditions (Figure 1). The expression and enzymatic activity of the SPS gene have been observed in diverse non-photosynthetic organs such as germinating seedlings, leaves during the sink-to-source transition, and the pollen of immature inflorescences, where Suc biosynthesis and allocation also occur, to support the formation of polymeric compounds and the accumulation of carbohydrates in response to environmental stresses, including water deficits and extreme temperatures [63,65,66,67,68,69,70,71].

## 3. Plant SPS Isoforms and Their Role in Sucrose Biosynthesis, Tissue Allocation, and Plant-Beneficial Microbe Interactions

**SPS** is encoded by a small multigenic family of three to seven gene copies in angiosperms [72,73,74]. Different SPS genes may exhibit redundancy on the tissue-specific pattern of expression in a plant species [21,69,73,75]. Certain SPS genes within a plant may play particular physiological roles, and thus may be expressed in specific tissues, at precise diurnal periods or in concrete developmental stages [63,69,71,75]. During the germination of rice (*Oryza sativa*) seeds, the expression of starch hydrolytic enzymes and SPS is coordinated, suggesting that the harmonic activity of both enzymes drives Suc biosynthesis from starch to support embryo development; in opposite circumstances, the expression of SPS genes has been observed in pollen, related to starch anabolism and accumulation during immature grain development [63,70,71].

*Arabidopsis thaliana* genes *AtSPS1* and *AtSPS2* are expressed in the floral nectaries, and their enzymatic products together with SWEET proteins constitute the nectar secretion mechanism; SPS enzymes in the nectary parenchyma drive starch-derived Suc synthesis, which is then exported to the extracellular space via SWEET proteins. Subsequently, Suc is cleaved by apoplastic invertase, elevating the osmotic potential and latterly the water flow [76]. Some SPS genes are expressed preferentially or exclusively in heterotrophic tissues such as roots, flowers, and fruits in model plants such as *A. thaliana* [75] or tobacco [21], suggesting that SPS is also a key enzyme of Suc synthesis in non-photosynthetic tissues.

The role of SPS and Suc biosynthesis in mutualistic interactions has been substantially studied in the rhizobium–legume symbiosis between *Medicago sativa* and *Sinorhizobium meliloti* [77,78]. Three genes encode the different SPS isoforms in *M. sativa MsSPSA*, *MsSPSB*, and *MsSPSB3*. When *M. sativa* was inoculated with *S. meliloti*, *MsSPSB* and *MsSPSB3* were exclusively expressed in leaves, while *MsSPSA* expression was enhanced in nodules, and their protein isoforms responded differentially to allosteric regulation by Glc-6-P and Pi. The study of SPS, SuSy, and SPP in WT and Fix^−^ mutant nodules suggested that SPS activity plays an important role in the physiology of the nodule and in the mobilization of carbon towards the symbiotic microorganism [77]. Further studies in *M. sativa* defined that SPSB controls sucrose biosynthesis in leaves, while SPSA is crucial for the maintenance of the regulatory cycles of sucrose biosynthesis/breakdown from sucrose and starch catabolic products [78]. These results suggest that SPS could be involved in the modulation of the C amount provided to the symbiont through the Suc synthesis in the nodules, and that the nodule-induced SPS gene expression from a particular phylogenetic family is specifically regulated in response to the interaction of the plant with mutualistic microorganisms [77,78]. Suc transported to the nodules is primarily catabolized in the vascular tissues, and the hexoses obtained are then transported to the central part of the nodules, where they are routed to starch biosynthesis. A later canalization of C from starch to Suc is required to maintain a stable carbohydrate/energy supply for the optimum functioning of the nitrogen-fixing nodule. A correct supply of carbohydrates is needed for N_2_ fixation and N assimilation through the glycolytic or the oxidative pentose phosphate pathways to obtain phosphoenolpyruvate (PEP), oxaloacetic acid and L-malate or α-ketoglutarate to fuel nitrogen fixation and the assimilation of ammonia [79].

The modulation of the photosynthetic rates in mycorrhizal plants may imply the regulation of SPS gene expression and enzymatic activity to establish a Suc distribution that meets the plant’s demands and allows the carbon supply to the AMF. Nonetheless, experimental evidence on the role of SPS in controlling carbon partitioning in the AM symbiosis is not conclusive: for example, no differences in SPS activities in leaves from mycorrhizal and non-mycorrhizal plants were found in *C. sativus* under different P levels [18], while SPS activity increased in the leaves of mycorrhizal *Poncirus trifoliata* [80]. The latter study revealed a correlation between the increase in the net activity of Suc metabolic enzymes and the soluble sugar content in the leaves of AM plants, which was even more pronounced when plants were exposed to drought conditions, suggesting that SPS modulates Suc production in the source tissues to support the demand for C during symbiosis, but also to maintain the osmolyte accumulation under water-limiting conditions [80].

The increase in P in the plant tissues promoted by AM may indirectly impact SPS modulation and Suc biosynthesis, since Pi is fundamental to the translocation of triose-Ps from the chloroplast [81], and it also regulates the enzymatic activity of SPS by covalent modification and allosteric modulation [62]. Therefore, approaches to studying Suc biosynthesis in mycorrhizal plants must consider the availability and dynamics of P in relation to the transcriptional and post-transcriptional regulation of SPS and the sink stimulation exerted by the mycorrhizal establishment.

Thus, the reported studies suggest that the genetic elements and molecular mechanisms regulating SPS isoforms respond to the mutualistic interactions. Indeed, the synthesis of Suc is modulated in areas where the exchange of nutrients between the symbionts takes place during the plant–microbe interaction and determines the amount of carbon that is provided by the plant to the microorganism.

**Figure 2:**
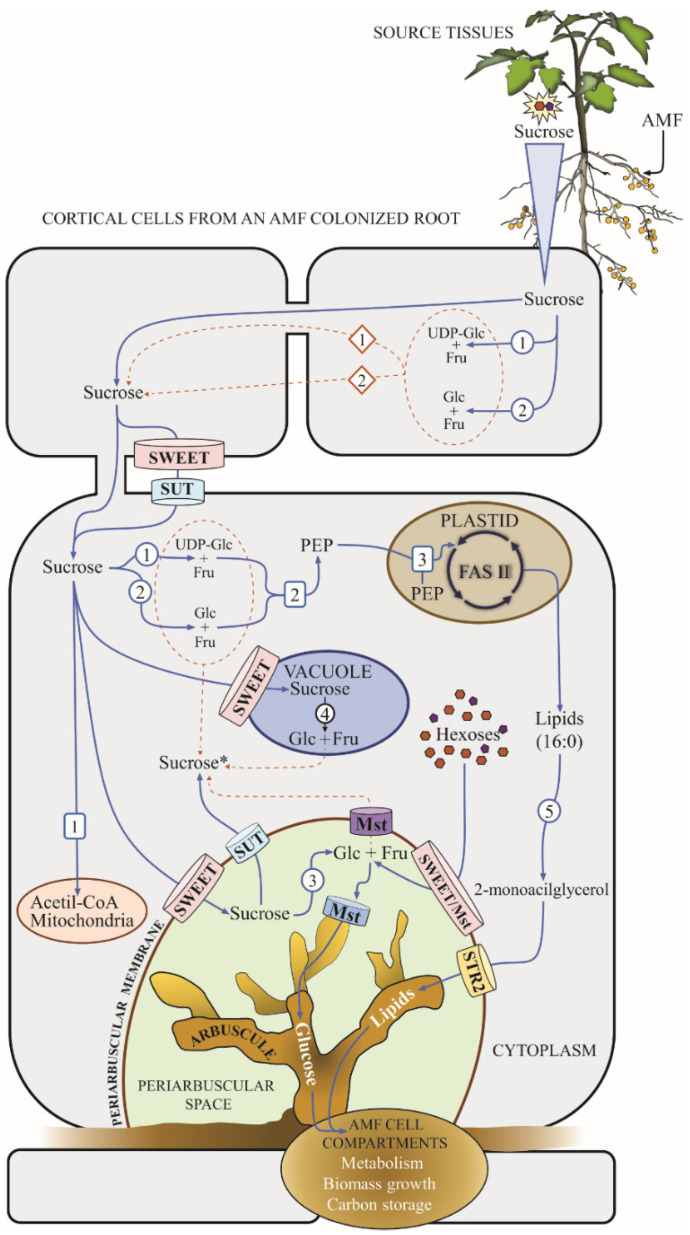
Carbon flux to the cortical root cells during arbuscular–mycorrhizal interactions. Photosynthates flow through the mycorrhizal plant from the leaves to the arbusculated cortical cells in the roots. The catabolism of Suc in the arbusculated and other cortical cells close to them promotes Suc mass flow and enables the translocation of hexoses, Suc, and lipids to the periarbuscular space towards the fungal arbuscule, imposing a carbon sink (Updated from Wipf et al., 2019; Roth and Paszkowski, 2017; and Manck-Götzenberger and Requena, 2016 [8,34,39]). Blue arrows trace the current carbon flow routes, the discontinued orange arrows showthe “futile” cycles of sucrose catabolism and synthesis, the colored barrels designate the carbohydrate transporters. Enzymes are indicated in numbered circles as: (1) Sucrose synthase; (2) Neutral invertase; (3) Apoplastic invertase; (4) Vacuolar invertase; (5) Glycerol-3-phosphate acyl transferase. Numbers in rounded rectangles denote specific metabolic pathways: (1) Suc as a source for aerobic respiration; (2) Glycolytic pathway to render phosphoenolpyruvate; (3) lipid synthesis mediated by the plastid Type I FAS molecular complex. Diamonds indicate potential carbon fluxes as: (1) symplastic and (2) apoplastic routes of hexoses entry to sink cells. Sucrose * indicates the sucrose biosynthesized in the arbusculated cell.

The scheme in Figure 2 integrates the current knowledge on carbon allocation to root cells during plant–AMF interactions. We propose that Suc biosynthesis occurring in colonized cells may control the carbon supply to the AMF in the form of carbohydrates and lipids.

## 4. Sucrose Transporters and Sucrose Mobilization in Mycorrhizal Plants

To achieve carbon loading in non-photosynthetic tissues, Suc synthesized in mesophyll cells reaches the apoplast adjacent to the phloem, where phloem loading is mediated by **SUT** [22]. The SUT proteins form a small three clade family divided into types I, II, and III; SUT types I and II are found in the phloem tissues and they import Suc into the phloem for the mobilization of carbon from the source to the sink tissues. The transporters of the type III clade are located in the tonoplast membrane where they transiently load Suc into the tonoplast [82].

A semiquantitative RT-PCR transcriptional analysis of SUT transporters in tomato (*S. lycopersicum*) plants colonized by *Glomus caledonium* or *G. intraradices* estimated the down-regulation of *SlSUT1* in the source tissues of mycorrhizal *S. lycopersicum*, and its expression was not influenced by the availability of P, but it was related to the presence of the AMF in the roots [83]. Further quantitative RT-PCR (qRT-PCR) studies in *F. mosseae* colonized *S. lycopersicum* plants revealed an up-regulation of *SlSUT1* (type I), *SlSUT2* (type II)*,* and *SlSUT4* (type III) in the leaves. While *SlSUT1* and *SlSUT4* were up-regulated in the roots, *SlSUT2* was not [84].

In *M. truncatula*, the increased expression of *MtSUT1-1*, *MtSUT2,* and *MtSUT4-1* (orthologous to *SlSUT1*, *SlSUT2,* and *SlSUT4*, respectively) was described by qRT-PCR in the leaves and roots of plants grown at low P concentrations (NaH_2_PO_4_, 0.13 mM); the expression of those genes in the leaves was even higher under *Rhizophagus* sp. colonization, suggesting that AMF induces carbon flux from the source tissues to the phloem [85]. The roots also showed more *MtSUT1-1* transcript accumulation in low P when plants were mycorrhized; however, *MtSUT2* and *MtSUT4-1* were similar in mycorrhizal and non-mycorrhizal plants [85].

According to the studies reported above, the *S. lycopersicum* and *M. truncatula* SUT orthologous from the same divergent clades exhibit similar expression patterns in the source and sink tissues in response to mycorrhizal colonization. While genes from the three SUT types were up-regulated in the leaves of both plant species, only type I and III transporters were transcriptionally up-regulated in mycorrhized roots. Thus, we propose that the enhanced expression of SUT orthologous genes in the source and sink tissues in response to mycorrhizal colonization in angiosperms of divergent lineages is the result of a conserved molecular mechanism to supply carbon to AMF in which the Suc flow mediated by SUT transporters is a common trait in higher plants.

The enhanced SUT expression observed in mycorrhizal *S. lycopersicum* correlated with Suc and Fru accumulation and the decrease in Glc in the roots [84]. These results suggest that the carbon delivered to the roots as Suc is catabolized to generate Glc that is preferably used to supply carbon to the AMF, thus contributing to Fru accumulation in the root tissues [84]. In *M. truncatula*, lower concentrations of Suc, Glc, and Fru in the leaves of mycorrhizal plants was proposed to be a consequence of higher sugar transport activity in source tissues [85].

The heterologous expression of the spinach (*Spinacia oleracea*) SUT1 transporter gene (*SoSUT1*) fused to the constitutive CaMV 35S promoter led to an increase in the colonization capacity of *R. irregularis* in *S. tuberosum*, despite high P levels in the soil. This high P concentration strongly inhibited AMF colonization in the wild-type genotype and in a silenced *SUT1* mutant that was also tested under the same conditions, supporting the claim that the carbon supply exerted by the source tissues controls AMF colonization [86]. Plants inoculated with *R. irregularis* at a low P content showed similar levels of colonization in the wild type and the overexpressing and the silenced *SUT1 S. tuberosum* mutants; therefore, it was hypothesized that when phloem loading is impaired due to *SUT1* silencing, plants tend to prioritize carbon delivery to sustain the mycorrhizal symbiosis. In agreement with this hypothesis, mycorrhized silenced *SUT1* plants showed a lower biomass accumulation [86].

In grafting experiments in which *S. lycopersicum SUT2*-silenced mutants and wild-type genotypes were combined, Bitterlich et al. (2014) demonstrated a reproducible increase in root colonization by *F. mosseae* or *R. irregularis* when inoculated independently in silenced *SlSUT2* root stocks, indicating a root-specific function of SUT2 in the carbon flux to AMF. Furthermore, the positive growth response to AMF colonization was abolished in *SlSUT2* antisense plants [38]. Remarkably, the SUT2 transporter was specifically immunolocalized in the periarbuscular membrane in cortical cells, suggesting that SUT2 transports Suc from the periarbuscular matrix back to the cytoplasm of the plant cell. Thus, SUT2 exerts an important influence on carbon distribution between symbionts in mycorrhizal associations, regulating the amount of Suc in the periarbuscular space and controlling the carbon supplied to the fungal symbiont [38]. The phenotype of *S. lycopersicum SUT2*-silenced plants can be partially rescued by the exogenous application of brassinosteroids, suggesting that the biosynthetic/signaling pathway of this plant growth regulator is linked to the Suc–carbon partitioning pathway, mediated by SUT2. Therefore, it is possible that brassinosteroids may be involved in carbon distribution to AMF during the arbuscular mycorrhizal interaction [38,87].

The most recently discovered plant carbohydrate transporter proteins are the **SWEET transporters** [23]. They are encoded by a multigenic family that clusters four subgroups classified by their affinity for carbohydrates. In *S. lycopersicum*, the four groups are subsequently separated into Class I transporters that mediate Glc and Fru transport, and Class II transporters, which have a higher affinity for Glc and Suc [88]. SWEET transporters function as Suc exporters, releasing Suc to the apoplast, and their function is essential for phloem loading [89]. Several events of plant development such as the embryonic and reproductive tissue development in *A. thaliana*, *Glycine max*, and *Petunia axillaris* have been related to the carbohydrate transport activity of SWEET proteins [90,91,92].

Increasing evidence indicates that SWEET transporters play major roles in the mutualistic and pathogenic interactions of plants with microorganisms [39,93,94,95]. The differential regulation of some SWEET genes has been demonstrated. For example, the genome of *Lotus japonicus* contains 13 SWEET codifying genes. The transcriptional analysis of this gene family during symbiosis revealed that only *LjSWEET3* is up-regulated in nodules formed by *Mesorhizobium loti*, and its expression was also increased in roots colonized by *R. irregularis* [94]. The expression level of the 35 SWEET genes in the *S. tuberosum* genome was analyzed in roots inoculated with *R. irregularis* in three temporally defined stages of mycorrhizal development, and 22 of these genes were differentially expressed in at least two of the three stages studied [39]. The study led to the identification of three SWEET genes from divergent clades in the transporter family that were induced by the mycorrhiza. The promoter of these three genes was subsequently fused to the β-glucuronidase gene to characterize their expression in non-mycorrhized and mycorrhized *M. truncatula* plants. In all cases, the reporter gene expression was specifically detected in the cortical cells containing arbuscules. In particular, the promoter of *StSWEET2a*, a putative Suc transporter, controlled the expression of the reporter gene only at the root apex of non-mycorrhized plants. According to these authors, the induced specific expression of *StSWEET2a* in the arbusculated cortical cells supports the claim that Suc is an important source that helps the carbon demands of the fungal symbiont in arbuscular mycorrhizal interactions to be met [39]. This transcriptional analysis of SWEET genes in *S. tuberosum* showed that *StWEET1a*, *StSWEET1b*, and *StWEET7a* are genes that are up-regulated in mycorrhized plants [39]. Their putative orthologs in *M. trucatula*, *MtSWEET1b*, and *MtSWEET6* also showed higher expression levels in mycorrhizal plants [96]. Remarkably, the simultaneous colonization of *M. truncatula* by *R. irregularis* and *Ensifer meliloti* in a tripartite association led to a reduction in mycorrhizal colonization, the down-regulation of *MtSWEET1b* and *MtSWEET6* expression in the roots, and the up-regulation of *MtSWEET15d* in the root nodules. Accordingly, the authors concluded that *MtSWEET1b* and *MtSWEET6* play a key role in the specific carbon transfer to the AMF [96]. An et al. (2019) further characterized this mycorrhiza-upregulated *MtSWEET1b* as a Glc transporter located in the periarbuscular membrane [97]. Phylogenetically, *MtSWEET1b* shares its origin with *MtSWEET1a*, as both are homologous to the *A. thaliana* gene *AtSWEET1*, a bidirectional uniporter facilitator of Glc that is highly expressed in flowers and pollen tubes, but with a weak expression in the roots [97,98]. There are at least two SWEET genes homologous to *AtSWEET1* in the genome of plants that belong to both lineages of angiosperms such as *O. sativa*, *L. japonicus*, and *S. tuberosum*, and as reported for *M. truncatula*, at least one of those genes showed an up-regulation in mycorrhized plants [97].

As proposed for SUT transporters, the activity of the SWEET genes described in AM points to a common molecular mechanism for carbon delivery to the fungus in divergent clades of angiosperms, and they appear to play an essential role in plant–microbe interactions. Moreover, the lack of mycorrhizal-responsive homologs of SWEET transporters in *A. thaliana* suggests that, besides the inability for proper molecular signaling with AMF, non-mycorrhizal plants may have lost the genes required to maintain an efficient carbon efflux to the fungal symbiont (Figure 2). In this regard, it was recently reported that non-mycorrhizal plants are unable to interact with AMF due to the loss or pseudogenization of key genes essential for plant-microbial signaling [99].

In addition to the sugar transporters belonging to the SUT and SWEET families, **monosaccharide transporters** have also been associated with carbon partitioning to the fungal symbiont in arbuscular mycorrhizal interactions. The first experimental evidence for the induced expression of an *M. truncatula* hexose transporter (*Mtst1*) in the roots of *M. truncatula* and *M. sativa* colonized by an AMF was presented by Harrison (1996). She also reported the induced expression of *Mtst1* in *Medicago* roots colonized by *G. versiforme* and located its transcripts in arbusculated cortical cells by in-situ hybridization [35].

The *S. lycopersicum* transporter *SlSFP7* (formerly named *LeST3*) was subsequently identified and characterized by García-Rodríguez et al. (2005) as a hexose transporter of the major facilitator superfamily [100,101]. Its expression level increases in the source tissues in plants colonized by different AMF and by the phytopathogen *Phytophthora parasitica*, while the level of expression remains constant in the roots. The authors suggested that the up-regulation of this monosaccharide transporter in the leaves is the result of the increased carbon demand imposed by the pathogenic or mutualistic fungal interaction, and its function is to mobilize hexoses from the source tissues to the roots [100]. Ge et al. (2008) also compared the expression of *SlSFP7* in plants colonized by AMF. They described that when *S. lycopersicum* roots were colonized by *G. intraradices*, the expression level of *SlSFP7* increased in the leaves and roots. However, in plants colonized by *G. caledonium*, the expression levels of this transporter were reduced compared to the control plants, in disagreement with the former hypothesis of *SlSFP7* responding to fungal interactions independently of its parasitic or mutualistic behavior, as proposed by García-Rodríguez et al. (2005) [100]. Remarkably, *SlSFP7* was the only sugar transporter evaluated by Ge et al. (2008) that showed a transcriptional response to mycorrhization. The contrasting response in the expression of *SlSFP7* in *S. lycopersicum* plants colonized by different AMF species was interpreted as the recruitment of different molecular elements in a single plant species by different AMF species to obtain a carbon supply [83].

The previous studies suggested that different AMF species may exert different levels of carbon sink strength, and thus the level of stimulation of carbon allocation to the symbiotic interphase. A fine-tuned regulation of the expression of plant carbohydrate transporters was also described as involved in the carbon allocation towards the fungal symbiont [85]. Moreover, positive growth responses in mycorrhizal plants showed an up-regulation of SWEET, while non-cooperative mycorrhizal interactions did not [102].

Based on these results, we propose that the ability of AM to impact plant carbon allocation and establish itself as a sink depends in part on the ability of AMF to stimulate the expression and activity of plant carbohydrate transporters, resulting in the amount of carbon received from the host. Consequently, a mycorrhizal interaction that promotes sink stimulation will promote plant growth.

Increased expression of the monosaccharide transporter *ZmMST1* was detected in maize roots colonized by *G. intraradices*. The up-regulation of this gene was observed under conditions of P scarcity and was related to an increase in the concentration of soluble sugars in roots, supporting the role of this transporter in the carbon allocation to AMF [103].

In summary, an analysis of sugar transporters in AMF-colonized plants suggests that to sustain carbon uptake by AMF, sugar is exported to the periarbuscular space through molecular mechanisms controlled by SWEET proteins in the periarbuscular membrane. SWEET proteins have also been proposed as active controllers of the carbon supply by acting as cytoplasmic importers of Suc/hexoses released to the periarbuscular space. The common expression patterns of orthologous genes observed in different model plants upon AMF colonization support the hypothesis of a common molecular mechanism that supplies carbohydrates to the symbiont in vascular plants, in which SUT, SWEET, and monosaccharide transporters have a common role.

## 5. Mycorrhizal Symbiosis and Sucrose Catabolism in Plants

The genes encoding the SuSy and invertase enzymes are present in small families in plant genomes [104,105]. **Plant invertases** are located subcellularly in the cytoplasm, mitochondria, chloroplast, vacuole, and in the cell wall [105]. It has been proposed that in arbuscular mycorrhizal interactions, cell wall invertases, also named apoplastic invertases, hydrolyze Suc in the periarbuscular space and generate the hexoses that will be taken up by AMF. In this regard, promoter analyses and in-situ hybridization studies have revealed the expression of the *S. lycopersicum LIN6* promoter activity, which encodes an apoplastic invertase in arbusculated cells [36]. *LIN6* transcription was also up-regulated in response to environmental stresses, mechanical stimuli, and by pathogen infection [106].

The experimental evidence obtained for *S. lycopersicum* and *S. tuberosum* has led to recent models that describe carbon transfer from the host plant to the fungal symbiont, where apoplastic invertases directly generate hexoses delivered to AMF [36,37,39]. Vacuolar invertase expression is also induced in *Phaseolus vulgaris* roots colonized by *G. intraradices* [107], while the specific enzymatic activity of cytosolic invertases increased in *G. max*, colonized by *G. mosseae* compared to other types of invertases [108]. This supports the claim that the regulatory cycles of Suc biosynthesis and catabolism in subcellular compartments participate in carbon allocation to AMF during mycorrhizal interaction (Figure 2).

Tobacco plants overexpressing a yeast-derived invertase under the constitutive 35S promoter were inoculated with *G. intraradices*. Heterozygous lines with different increased invertase levels in the leaves displayed a higher accumulation of hexoses in their source leaves, but no increase in invertase activity or hexose accumulation was observed in the roots, regardless of the activity levels achieved in leaves. The plant lines with higher invertase activity showed lower hexoses content in the roots, together with the up-regulation of the pathogenesis-related (PR) genes *PAR1, PR-Q*, and *PR-1b* in the leaves, and reduced levels of root mycorrhization. These results indicated that hexoses accumulated in the leaves activated defense mechanisms potentially with a negative effect on AMF; moreover, they suggest that changes in carbohydrate metabolism in shoots influence the establishment of mycorrhizal interaction in the roots, and that this influence is not exclusively determined by the carbon supply to roots [109]. Interestingly, yeast invertase expression specifically targeted to the tobacco root system increased hexose accumulation, but it did not affect AM colonization [110]. Similarly, the transformed roots of *M. truncatula* expressing apoplast-, cytosol-, or vacuolar-located yeast-derived invertases accumulated hexoses, but this did not impact mycorrhization [110]. Thus, the authors concluded that the increase in hexose levels did not significantly impact the symbiosis physiology, and that the invertase-controlled carbon supply to the fungus is not a limiting factor. This suggests the existence of other controllers of carbon allocation in the roots [110], as the mechanism previously described regarding SUT transporters [38].

Similar to invertases, **Suc synthases** (**SuSy**) are encoded by a small family of genes in most plant species. In *M. truncatula*, five genes encoding SuSy have been identified in the genome, but only one, *MtSucS1*, was induced in mycorrhizal roots with *G. mosseae*. The fusion of specific regions of *MtSucS1* to the *gus*Aint reporter gene led to the localization of the chimeric protein in arbusculated cells, but also in the adjacent cells [111]. An analogous pattern of expression of SuSy transcripts in the roots of *P. vulgaris* colonized by *G. intraradices* was previously observed by in-situ hybridization [107]. To further analyze mycorrhizal physiology, *M. truncatula MtSucS1*-antisense lines were inoculated with *G. mosseae*. The arbuscules in the roots of these plants were early-senescent, did not reach complete differentiation, and colonization was impaired compared to the wild-type phenotype. This aberrant mycorrhizal phenotype was also accompanied by a reduced expression of mycorrhiza-induced plant genes, such as the Pi transporter *MtPT4*. This led to the conclusion that *MtSucS1* is essential to maintain normal arbuscular development and its influence in carbon distribution cannot be replaced solely by invertase activity [112]. Indeed, mycorrhizal colonization-induced genes encoding SuSy and invertases with different subcellular localization, including the LIN6 gene in *S. lycopersicum* [100]. It has been proposed that SuSy is an important element that generates Suc mobilization gradients to arbusculated cells by maintaining Suc cleavage in the cytoplasm to generate products that could be used to fuel the metabolically active colonized cell, or to export hexoses to the AMF during the interaction [100,107,111].

In summary, it is widely recognized that apoplastic invertase activity generates hexoses from Suc catabolism to be delivered to the fungal partner during mycorrhizal interactions; therefore, its function is to reduce the concentration of Suc in the periarbuscular space to maintain a constant Suc efflux to this zone, while the influx of Suc to the arbusculated cell is also maintained by SuSy and invertases located in other subcellular spaces such as the vacuole and the cytoplasm.

## 6. Lipids in Carbon Partitioning to the Arbuscular Mycorrhiza

Early strategies for the study of AMF carbon metabolism used transformed carrot (*Daucus carota*) roots inoculated under in vitro conditions with both symbionts placed in separated compartments [113,114]. The use of radiolabeled carbon substrates delivered to either compartment, containing the host root or the fungi, allowed the tracing of the carbon partitioning in the mycorrhizae by analyzing the pattern of storage and structural labeled molecules. This approach revealed that the carbon transferred as carbohydrates from the host to the AMF was metabolized to triacylglycerols (TAG) in the intraradical mycelium, and then translocated to the extraradical mycelium to constitute the major fungal carbon storage, which is mainly found in spores, vesicles, and hyphae [114]. The analysis of asymbiotically germinating spores indicated that the gluconeogenesis and the glyoxylate pathway were both active in germination tubes, but evidence to support fatty acid (FA) synthesis at this stage was lacking. Thus, it was speculated that FA synthesis would be restricted for membrane synthesis [115], and this limiting condition would prevent the formation of new propagules, explaining the obligate biotrophy of the AMF [116]. Additional studies have indicated that hexoses acquired by AMF are metabolized in the intraradical mycelium to form triacylglycerol (TAG) and glycogen, rather than trehalose. According to this, TAG and glycogen would be subsequently translocated to the extraradical mycelium to maintain the biosynthesis of storage and structural polymers such as chitin [117].

The idea of FA de novo synthesis occurring in the intraradical mycelium from carbohydrates and its translocation to the extraradical mycelium prevailed until the sequencing and publication of the *R. irregularis* genome and transcriptome [118,119]. Wewer et al. (2014) found sufficient evidence to indicate that *R. irregularis* have the molecular mechanisms to drive FA oxidation and elongation/desaturation, but not for the presence of the multidomain type I FAS, responsible for the de novo biosynthesis of FA [29]. De novo FA biosynthesis, a central pathway in the primary metabolism, is controlled by type I FAS, a macromolecular complex that controls C16:0 and C18:0 biosynthesis in fungi and mammals [120,121]. The lack of de novo FA biosynthesis machinery in AMF may explain their obligate biotrophy, and supports the idea that palmitic acid (C16:0) is supplied by the host during mycorrhizal interaction [29]. A few years after this scenario was proposed, Jiang et al. (2017) and Luginbuehl et al. (2017) provided new evidence to demonstrate that lipids are synthesized de novo in the host plant and allocated to the AMF. They also confirmed that carbon supply to the AMF as lipids is essential to achieve a normal mycorrhizal phenotype in *M. truncatula* [27,28]. Moreover, the absence of the type I FAS molecular complex in the AMF as *Gigaspora margarita* and *R. clarus*, indicated that the inability to synthesize lipids de novo is a common feature among AMF [30,122].

Recently, new studies on the regulation of FA biosynthesis are shedding light on the regulation of the mycorrhizal functioning. WRINKLED transcription factors from the AP2-EREBP family (APETALA2-ethylen-responsive element binding protein) control FA biosynthesis in *Arabidopsis* [40]. In *L. japonicus*, *CTT Motif-Binding Transcription Factor1* (*CBX1*), a *WRINKLED1* homologue, has been described to activate the mycorrhizal-inducible genes encoding the phosphate transporter 4 (*LjPT4*), H+ -ATPase (*LjHA1*), and *RAM2* [42]. Similarly, *M. truncatula* transcription factors WRI5, WRI5/Erf1, and WRI5/c regulate the expression of *STR* and *MtPT4* genes, and are involved in the formation of the periarbuscular membrane [41].

Rich et al. (2021) demonstrated that the transcriptional activation of FA biosynthesis is a common response to AMF in angiosperms and lower plants such as Bryophytes [33]. They showed that the liverwort *Marchantia palacea* can transfer lipids to *R. irregularis* and that the orthologous genes *RAM2* and *STR*/*STR2*, are induced in mycorrhizal thalli under the control of a WRINKLED transcription factor (*MpaWRI*) [33].

These data indicate the existence of a common molecular program shared between Bryophytes and angiosperms to activate lipid transfer to the AMF could be present in primitive plants, prior to the divergence between Bryophytes and vascular plants. This supports the hypothesis that AM establishment was a key factor during the early stages of Earth colonization by plants, 450 million years ago [31,32,33].

The discovery of the lipid auxotrophy in AMF led to the improvement of axenic culture techniques. By adding myristate or a mixture of myristate and palmitic acid to culture media, Sugiura et al. (2020) managed to germinate in vitro spores of *R. irregularis* and *G. margarita*, producing masses of fungal hyphae that formed functional spores, so that for the first time the spores generated in axenic cultures preserved the potential to colonize roots [123]. Additionally, adding strigolactones, jasmonic acid, and organic nitrogen to the culture media supplemented with myristate produced an even higher number of spores; moreover, the so-obtained spores promoted plant growth in symbiotic culture systems [124]. These advances in AMF in vitro axenic cultivation paved the way for a further improvement of AMF mass production for agrobiotechnological applications, but they have also opened new possibilities for studies on AMF development, genetics, and metabolism without the need or influence of a host.

Despite these advances, the significance of host carbon transfer as lipids or as carbohydrates to the fungus in the terms of host growth and development during mycorrhizal symbiosis remains an open question. Transcriptomic evidence has shown that the AMF- inoculated roots of *G. max* plants with different levels of colonization and plant growth promotion increased the expression of genes related to sugar transport and Suc catabolism only when plant growth was promoted, and Suc, Glc, and Fru content increased [102]. In the same study, *RAM2* and *STR/STR2* were up-regulated in colonized plants, independently of the inocula used and the growth promotion achieved. The contrasting reconfiguration of sugar compared to lipid gene expression suggested that growth enhancement in plants is strongly related to the stimulation of sugar transport and Suc catabolism in the host plant [102].

Glycolysis also participates in the regulation of sugar accumulation in plant organs, contributing to carbon distribution. PEP can be used to fuel lipid biosynthesis controlled by the plastid FAS molecular complex. Thus, plant lipid biosynthesis occurs within plastid organelles, where the Suc catabolism and hexose oxidation are essential steps to provide a carbon source for de novo lipid biosynthesis [125,126]. The disruption of the glycolytic pathway by the antisense silencing of cytosolic Triose-Phosphate Isomerase (TPI, EC 5.3.1.1) caused an increase in Suc, Glc, Glc-6-P, Fru, fumarate, and isocitrate in the roots of silenced TPI genotypes, which was accompanied by an increased in the total lipid concentration [127]. In parallel to PEP from glycolysis, alternate Suc catabolism sub-products, such as Glc-6-P, can be translocated into the plastids and can subsequently be used to synthesize PEP (Figure 2) in the stroma by plastidic glycolysis to support lipid biosynthesis [126,128].

Consistent with the increases in Suc content reported in arbuscular mycorrhized roots in several plant models [84,85,102], a higher concentration of Suc in arbusculated cortical cells has been shown by metabolite profiling after laser capture microdissection [129]. However Suc accumulation in *Arabidopsis*, a non-mycorrhizal model, mediates KIN10 inactivation, a negative regulator of WRINKLED1 (WRI1), which causes FA accumulation, suggesting that sugar/Suc homeostasis plays a regulatory role in lipid biosynthesis [130,131]. This suggests that mycorrhization can trigger the accumulation of Suc in roots and arbusculated cells, as a mycorrhiza-derived signal, impacting the lipid biosynthetic pathway (Figure 2). Suc content seems to be dynamically modified by the phenology and physiology of the mycorrhizae as indicated by studies on root Suc content showing that differences between non-mycorrhizal and mycorrhizal plants depend on plant age and the progression of mycorrhizal colonization [37]. How this dynamic regulation affects lipids is yet to be addressed.

## 7. Conclusions

Several studies have reported that AM colonization has a strong influence on the carbon assimilation capacity of the plant, and on carbon partitioning and allocation towards the plant organs. The additional carbon sink imposed by the presence of AMF in the roots activates a molecular machinery that controls carbon partitioning, coordinating the activities of Suc biosynthesis, transport, and catabolism to generate carbon-based molecules that will be translocated to the AMF. The molecular elements involved in carbon transfer to AMF as carbohydrates and lipids, as well as the mechanisms of their regulation, appear to be conserved among plant lineages.

The role of Suc in the AM interaction is not limited to the plant primary metabolite and the carbon and energy source for carbon-based molecules to support AMF growth and development. Suc accumulation in plant tissues provides a cellular environment that promotes lipid biosynthesis by complex regulatory mechanisms. Therefore, it is plausible to consider that the Suc accumulation is a mycorrhiza-derived signal, promoted by the sink capacity of AMF, to control the host cell environment to achieve distinct goals: (1) to increase the hexoses content to become susceptible to be taken up by the AMF; (2) to increase the carbon supply to maintain the root metabolism and to generate carbon precursors to ensure lipid biosynthesis; and (3) to control the stability of key molecular elements to increase and maintain lipid biosynthesis and transport to sustain AMF lipid auxotrophy.

Fine-tuning the cost and benefits of the symbiosis is key for the plant. The re-incorporation of Suc from the periarbuscular space to the cytoplasm of arbusculated cells, driven by SUT proteins, supports the existence of symbiosis regulatory mechanisms to restrict the fungal growth to levels not detrimental for the plant. Although important advances have been achieved in the last few decades regarding the control of carbon partitioning in the mycorrhizal symbiosis, the elucidation of additional mechanisms will be useful to optimize the use of AMF inoculants for plant growth promotion.

## Data Availability

Not applicable.
